# Chinmo function in cockroaches provides new insights into the regulation and evolution of insect metamorphosis

**DOI:** 10.1371/journal.pgen.1011993

**Published:** 2025-12-26

**Authors:** Jorge Escudero, Judit Gonzalvo, Maria-Dolors Piulachs, Xavier Belles

**Affiliations:** Institute of Evolutionary Biology, CSIC-Universitat Pompeu Fabra, Barcelona, Spain; Cornell University College of Agriculture and Life Sciences, UNITED STATES OF AMERICA

## Abstract

Insect metamorphosis occurs in two main forms, hemimetaboly (simple) and holometaboly (complete), both regulated by hormonal and genetic pathways involving the transcription factors Krüppel homolog 1 (Kr-h1), Broad-Complex (BR-C), and Ecdysone-induced protein 93F (E93). The BTB–zinc finger protein Chronologically inappropriate morphogenesis (Chinmo), recently identified in the fruit fly *Drosophila melanogaster*, an holometabolan, as a larval state maintainer, was studied here in the German cockroach, *Blattella germanica*, an hemimetabolan. We also examined another BTB transcription factor, Abrupt (Ab), based on findings in another holometabolan, the red flour beetle, *Tribolium castaneum*, suggesting a cooperative role. We characterized *chinmo* expression in *B. germanica* and found sustained transcript levels during the N4 and N5 nymphal instars, followed by a marked decline at the final N6 instar. RNA interference (RNAi) knockdown of *chinmo* at N4 induced precocious metamorphosis two molts later, accompanied by reduced *Kr-h1* and elevated *E93* expression. Combined knockdown of *chinmo* and *E93* revealed that Chinmo primarily represses *E93*. Similarly, *ab* knockdown also triggered precocious metamorphosis, decreasing *Kr-h1* and increasing *E93* expression; double knockdown of *ab* and *E93* indicated that Ab primarily promotes *Kr-h1* expression. These results expand the MEKRE93 pathway by identifying Chinmo and Ab as additional regulators that help maintain the juvenile state in both hemimetabolan and holometabolan insects. Holometaboly likely evolved from hemimetabolan ancestors through the embryonic internalization of wing primordia into imaginal cells, which enabled the emergence of distinct larval forms. Key regulatory factors like Kr-h1, Chinmo, Ab, BR-C, and E93, already present in hemimetabolan lineages, were conserved and rewired in holometabolans. Crucial shifts in this evolutionary transition include Chinmo-mediated inhibition of *BR-C* and an inversion in the juvenile hormone effect on *BR-C*, from activation to repression.

## Author summary

Insect metamorphosis can be simple, as in cockroaches, where nymphs transform into adults, or complete, as in fruit flies, which pass through distinct larval, pupal, and adult stages. While it is known that certain factors coordinate these changes, we focused on a factor called Chinmo, known in fruit flies for maintaining the juvenile state. We wanted to see if it plays a similar role in cockroaches. We found that Chinmo is active during juvenile stages and declines just before adulthood. When we reduced its levels in young cockroaches, they underwent premature metamorphosis. This was linked to an increase in *E93*, a gene that promotes adult development. We also studied a related factor, Abrupt (Ab), and found that lowering its levels inhibited *Kr-h1*, a gene that helps maintain the juvenile state. This indicates that Chinmo and Ab help preserve the juvenile form in cockroaches by regulating key genes: Chinmo by inhibiting metamorphosis-promoting genes, and Ab by activating juvenile-maintaining ones. From an evolutionary perspective, our findings in cockroaches support the idea that complete metamorphosis in more derived insects evolved by reusing and rewiring regulatory networks already present in their less modified ancestors.

## Introduction

Insect metamorphosis is classified into two types: simple (hemimetabolan) and complete (holometabolan). In hemimetabolan insects, the juveniles, commonly known as nymphs, closely resemble the adults. Nymphs grow until they reach a critical size, at which point they undergo a final molt, becoming reproductively mature adults with functional wings. The adults do not molt further. The juveniles of holometabolans, commonly known as larvae, are distinct from the adults. The larva grows until it reaches a critical size, then molts into a pupa, an intermediate stage between the larva and the adult, and ultimately to an adult, which also ceases molting (reviewed in [1]).

The regulation of metamorphosis is primarily described as the result of interactions among three transcription factors: Krüppel homolog 1 (Kr-h1), which has antimetamorphic properties [2,3]; Broad-Complex (BR-C), which specifies the pupal stage [4–6]; and Ecdysone-induced protein 93F (Eip93F), commonly referred to as E93, which promotes adult morphogenesis [7]. These three factors are regulated by two hormones: 20-hydroxyecdysone (20E) and juvenile hormone (JH) (reviewed in [1,8]). In hemimetabolan insects, JH induces the expression of *Kr-h1*, whose corresponding protein maintains the nymphal state by repressing *E93*, a regulatory axis known as the MEKRE93 pathway [9]. In holometabolan species, this pathway is modified by the incorporation of BR-C, which governs the formation of the pupal stage [10].

This understanding remained largely unchanged until Truman and Riddiford [11] introduced a new player: Chinmo (short for Chronologically inappropriate morphogenesis), a factor that maintains the larval state in the fruit fly *Drosophila melanogaster*. Chinmo is a BTB-zinc finger protein initially identified in *D. melanogaster* as a regulator of temporal neuronal identity in the brain. When Chinmo is suppressed in larvae, early-born neurons prematurely adopt the identity of late-born neurons [12], and concurrently, *BR-C* expression is upregulated [13]. Following the work of Truman and Riddiford, subsequent studies confirmed the role of Chinmo in maintaining the larval state not only in *D. melanogaster* [14] but also in two other holometabolans: the red flour beetle *Tribolium castaneum* [15] and the fall armyworm *Spodoptera frugiperda* [16]. These findings established Chinmo as an important factor in maintaining the larval state in holometabolan insects. However, this raised a new question: could Chinmo also play a role in hemimetabolan metamorphosis?

We aimed to address this question using the cockroach *Blattella germanica* as a model species. Our initial efforts focused on identifying the *chinmo* homolog in this cockroach and characterizing its expression patterns in various tissues during the metamorphic transition. However, during the course of our study, a new report was published on the function of *chinmo* in *D. melanogaster* [14], which included a brief notice showing that RNAi targeting *chinmo* in the antepenultimate nymphal instar of *B. germanica* resulted in 43% of individuals undergoing precocious metamorphosis two molts later. Although this intriguing finding partially overlapped with our research project, it left several important questions unresolved, most notably, by what mechanism Chinmo depletion induced precocious metamorphosis in the cockroach. Therefore, we proceeded with our project to address this and other critical questions.

The work on the beetle *T. castaneum* [15] not only reported the role of *chinmo* in metamorphosis but also identified *abrupt* (*ab*), a gene encoding another BTB-domain transcription factor, as a co-regulator of this developmental transition. A key finding was that the simultaneous knockdown of *ab* and *chinmo* induced precocious metamorphosis symptoms more effectively than *chinmo* knockdown alone. Based on this evidence, we included *ab* in our investigation on *B. germanica*.

## Results

### *chinmo* expression displays a broadly parallel pattern across most tissues

**Fig 1:**
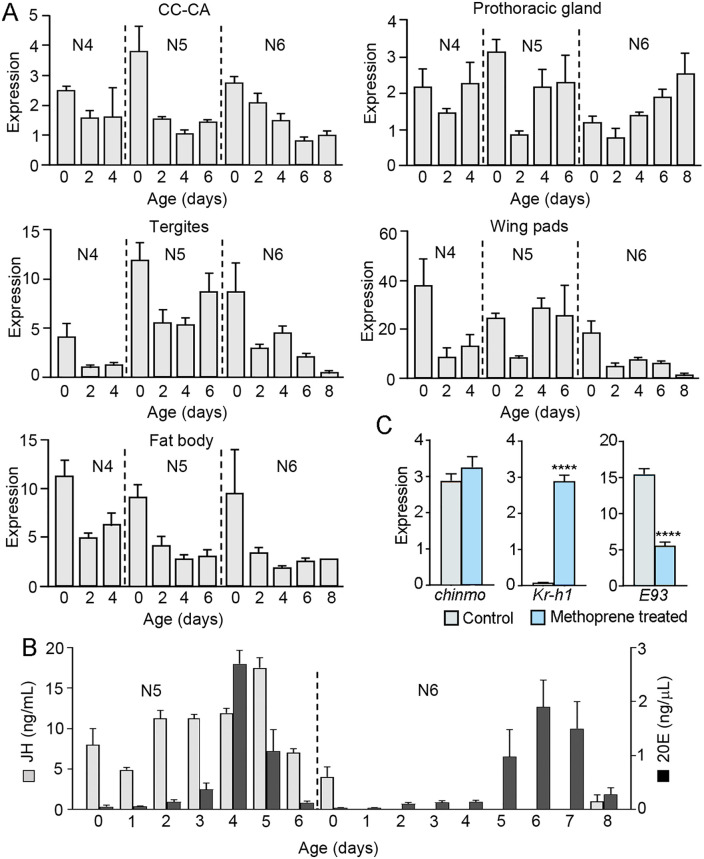
Expression pattern of *chinmo* in different tissues of *Blattella germanica* females, endocrine context, and effect of Methoprene on *chinmo* expression. (A) Expression of *chinmo* in the corpora cardiaca-corpora allata complex (CC-CA), prothoracic gland, tergites, wing pads, and fat body; the last three nymphal instars, the fourth (N4), the fifth (N5), and the sixth, and final (N6), were studied; within each instar, *chinmo* expression was measured at different days. (B) Concentration of juvenile hormone III (JH) and 20-hydroxyecdysone (20E) in the hemolymph of fifth and sixth nymphal instars (N5 and N6); JH data from [19]; and those of 20E from [20]. (C) Effects of Methoprene on *chinmo* expression in N6. A dose of 30 µg of Methoprene was topically applied on freshly ecdysed N6, and *chinmo* mRNA levels were measured two days later; the expression of *Kr-h1* and E93 was also measured. The results in (A) and (C) are indicated as copies of *chinmo* mRNA per 1000 copies of *Actin-5c* mRNA, and expressed as the mean ± SEM (n = 3-4). In (C), **** indicates significant differences (student’s *t* test) at p < 0.0001.

Using annotated *chinmo* sequences from a variety of insect species, we searched the *B. germanica* genome [17] and available transcriptomes [18] to obtain the complete coding sequence (CDS) of *chinmo*. We then analyzed *chinmo* expression during the final three nymphal instars: the fourth (N4), fifth (N5), and sixth (N6). For these analyses, we selected tissues involved in the production of key metamorphic hormones: the corpora cardiaca–corpora allata complex (CC-CA), which synthesizes JH, and the prothoracic gland (PG), which produces ecdysone that is then converted to 20E elsewhere. We also examined tissues that undergo marked morphological changes during metamorphosis, such as the epidermis (represented by the abdominal tergites) and wing pads (i.e. the pterotheca and wing primordia of the meso- and metathorax). Additionally, we included the fat body, a tissue that does not undergo significant metamorphic remodeling in *B. germanica*. The expression pattern of *chinmo* was broadly similar across all tissues: sustained levels during N4 and N5, with expression peaks around each molt, followed by a decline in N6. The only exception was the PG, where *chinmo* expression remained elevated and even increased during N6 (Fig 1A).

Comparison of *chinmo* expression with the profiles of JH and 20E concentration in the hemolymph (data from [19,20]) in the last two nymphal instars, N5 and N6 (Fig 1B) does not suggest obvious correlations, except that the decline of JH in N6 coincides with the decrease in *chinmo* mRNA levels. This suggests a cause-and-effect relationship, which we tested with an experiment with the JH analogue Methoprene. Thus, a dose of 30 µg of Methoprene was applied topically on freshly ecdysed N6, and *chinmo* mRNA levels were measured two days later. At the same time, we also measured the expression of *Kr-h1*, a JH-dependent gene, as a positive control of the Methoprene action, and *E93*, which is repressed by Kr-h1 [9]. The results showed that Methoprene effectively enhanced the expression of *Kr-h1*, but did not affect that of *chinmo*. At the same time, the dramatic increase in Kr-h1 led to a significant decrease in *E93* expression, consistent with the MEKRE93 pathway (Fig 1C).

### dsChinmo treatment in fourth instar nymphs induces precocious metamorphosis two molts later

**Fig 2:**
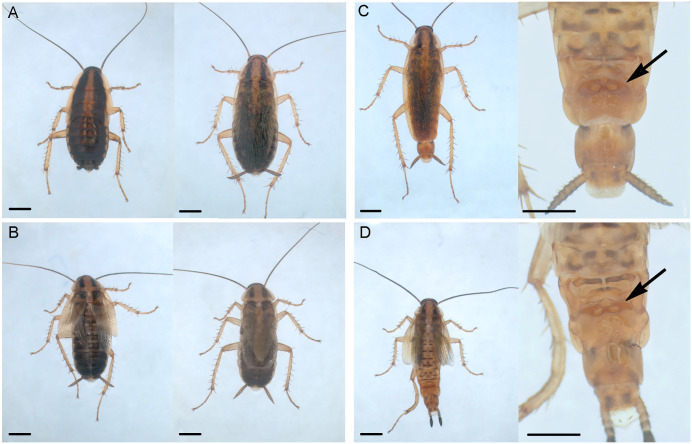
Precocious metamorphosis triggered by Chinmo depletion in *Blattella germanica.* (A) Control sixth instar female nymph (left) and control adult female (right). (B) Two precocious adult females, one with the membranous wings and tegmina wrinkled (left) and the other with these fully extended (right). (C) Control adult male (left) and detail of the abdomen showing the tergal gland (right, arrow). (D) Precocious adult male (left) and detail of the abdomen showing the tergal gland (right, arrow). Scale: 2 mm in all panels. Photos: Maria-Dolors Piulachs.

To investigate the role of *chinmo* in the metamorphosis of *B. germanica*, we depleted its mRNA levels using RNA interference (RNAi). Newly emerged fourth instar female nymphs (N4D0) were injected with 6 µg of dsChinmo, while control insects received 6 µg of a non-specific dsRNA (dsMock). Following treatment, the insects were monitored through subsequent molts. All dsMock-treated individuals (n = 32) molted normally to N5 and N6 (Fig 2A, left), and then to adults (Fig 2A, right), exhibiting no morphological abnormalities and following the expected timing. In contrast, all dsChinmo-treated individuals (n = 42) molted normally to N5, but then, instead of progressing to N6, precociously molted to adults. The precocious adults displayed external features typical of normal adult females, including coloration and morphology, but were noticeably smaller in size, similar to N6, and exhibited incompletely extended membranous wings and tegmina in 69% of cases (Fig 2B). Intriguingly, the membranous wings and tegmina were disproportionally shorter in precocious adults. Considering the tegmina, which do not wrinkle like the membranous wings, they barely reached three-quarters of the length of the body in the precocious adults. In contrast, in normal adults, they slightly exceed the body length. Three of the 42 dsChinmo-treated nymphs, initially misidentified as females, were later confirmed to be males. Their developmental pattern was consistent with that observed in females, undergoing a precocious imaginal molt following N5. The resulting males exhibited adult morphological traits, including the tergal gland, which is a male-specific structure [21] (Fig 2C). Similar to females, the male precocious adults were smaller in size; all three exhibited incompletely extended membranous wings and tegmina, which were disproportionally shorter with respect to the body length (Fig 2D).

### The ovaries of precocious adults initiate the adult maturation program, but it is defective

**Fig 3:**
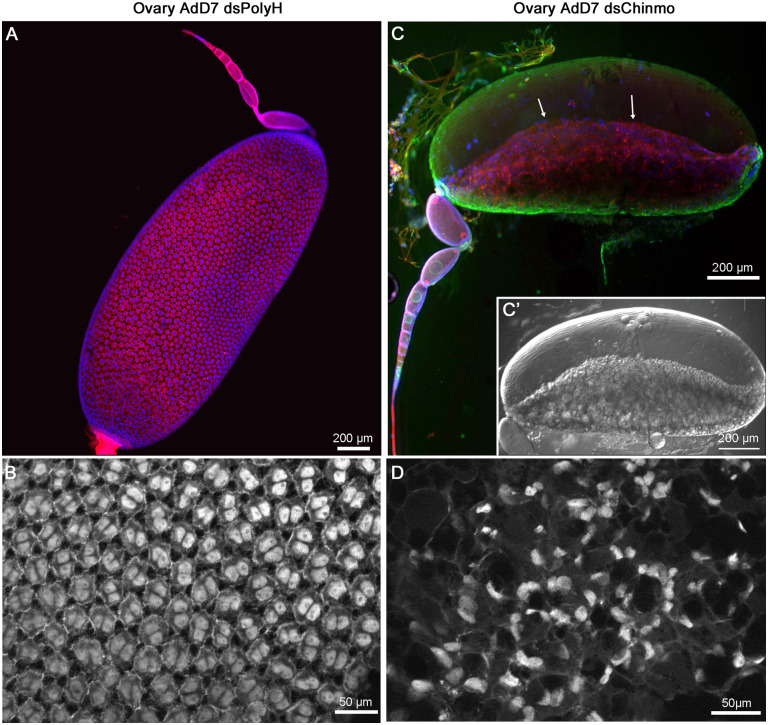
The ovary of a precocious adult obtained after Chinmo depletion compared with control females of *Blattella germanica.* (A) Ovariole of a 7-day-old control adult. (B) Detail of the follicular epithelium of the basal ovarian follicle (BOF) of an adult of the same age; the cells are binucleated and regularly distributed. (C) Ovariole from a 7-day-old precocious adult; the follicular epithelium does not cover the oocyte (arrows). (C’) The same BOF observed under differential interference contrast. (D) Detail of the follicular epithelium from C; the cells are disorganized, there are mononucleated and binucleated cells, with signs of disintegration. The F-actin microfilaments were stained with phalloidin-TRITC (red in A and C; white in B and D), and DNA was stained with DAPI (blue in A and C; white in B and D). In C, cell membranes were stained with WGA (green). Photos: Maria-Dolors Piulachs.

Seven days after the molt to precocious adult, we dissected three specimens to examine the ovaries and compare them to 7-day-old control adults (Fig 3A-3D). Notable differences were observed, particularly in the basal ovarian follicles (BOFs), whose length exhibited substantial variability, even within the same individual. From 37 ovarioles examined in three precocious adult females, we selected the 8 with the longest BOFs (1.49 ± 0.20 mm, n = 8) for further analysis. Although this length was significantly shorter than that observed in normal 7-day-old adult females (2.00 ± 0.03 mm, n = 10) [22], the follicular epithelium contained binucleated cells (Fig 3D), suggesting that they had initiated the adult maturation program. However, the most conspicuous BOF phenotype was that the follicular epithelium failed to completely envelop the oocyte (Fig 3C, arrows, and C’). In addition, it appeared disorganized and with cells often displaying pyknotic nuclei (Fig 3D). This contrasts with the well-structured follicular epithelium observed in control females BOFs (Fig 3B). This result raises the question of whether the incomplete deployment of the follicular epithelium is due to a lack of time, given that precocious metamorphosis skipped a nymphal instar, or to a direct role of Chinmo in regulating the mechanisms underlying follicular epithelium growth.

In *B. germanica*, follicular cells initially proliferate and subsequently switch to endocycles, a transition promoted by Notch signaling and repressed by the Hippo pathway, which downregulates Notch (N) [22,23]. To explore potential regulatory effects of Chinmo, we measured the expression of key genes of the above pathways in the ovaries of insects treated with dsChinmo at N4D0 and dissected at N5D5, an instar when follicular epithelium proliferation is active. The genes analyzed were *Epidermal growth factor receptor*, which interacts with both Notch and Hippo pathways; *yorkie*, a central downstream effector of the Hippo pathway; and *Delta* and *Serrate*, which encode ligands for the N receptor [22–24]. As shown in S1 Fig, although *chinmo* expression was significantly reduced in the ovaries, the expression levels of the selected genes from the Notch and Hippo pathways remained unchanged. These findings suggest that the impaired follicular epithelium growth observed in precocious adults is primarily due to insufficient time for full development. To further support this hypothesis, newly ecdysed adults (AdD0) were injected with 6 µg of dsChinmo (n = 6), while control insects received 6 µg of dsMock (n = 8), and ovaries were dissected at AdD7. As expected, ovaries from Chinmo-depleted adults were comparable to controls, with the follicular epithelium fully covering the oocyte in the BOF (S2 Fig).

### Chinmo depletion triggers an increase in *E93* expression and a decrease in *Kr-h1* expression

**Fig 4:**
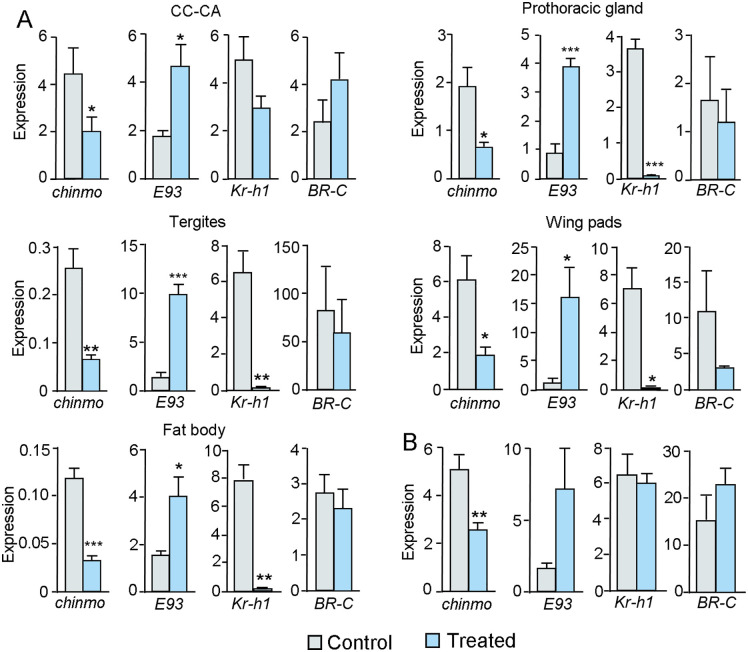
Effects of dsChinmo treatment on the expression of *chinmo* and other genes associated with metamorphosis in *Blattella germanica.* (A) Treatment in fourth instar female nymphs (N4). dsChinmo was applied in N4D0, and gene expression was measured at N5D4; the genes analyzed were *chinmo*, *E93*, *Kr-h1*, and *BR-C*, and the tissues examined were the corpora cardiaca-corpora allata complex (CC-CA), prothoracic gland, tergites, wing pads, and fat body. (B) Treatment in fifth instar female nymphs (N5). dsChinmo was applied in N5D0, and the expression of the same gene set was measured at N5D4 in wing pads. The results are indicated as copies of the examined mRNA per 1000 copies of *Actin-5c* mRNA, and expressed as the mean ± SEM (n = 3-5). Asterisks indicate statistically significant differences with respect to controls (* p < 0.05; ** p < 0.01; *** p < 0.001), calculated with the Student’s *t*-test.

The precocious metamorphosis induced by dsChinmo treatment prompted us to examine the expression of *E93*, *Kr-h1*, and *BR-C*, key genes involved in metamorphosis, as well as *chinmo* itself, to assess the efficacy of the RNAi treatment. dsChinmo was injected in N4D0, and gene expression was measured in the CC-CA, PG, tergites, wing pads, and fat body at N5D4, the day of the peak of 20E that triggers the molt to N6 [20]. The results revealed a significant depletion of *chinmo* expression across all tissues. Concurrently, *E93* expression was significantly upregulated, *Kr-h1* expression was significantly downregulated, whereas *BR-C* was unaffected (Fig 4A).

### dsChinmo treatment in fifth instar nymphs does not induce precocious metamorphosis

When dsChinmo was administered at N4D0, two molts were required before precocious metamorphosis occurred. This led us to investigate whether treatment in N5D0 could induce precocious metamorphosis after a single molt. To test this, newly emerged N5 (N5D0) were injected with 6 µg of dsChinmo, while control insects received 6 µg of dsMock. All treated individuals, including both dsMock-treated (n = 18) and dsChinmo-treated (n = 24) groups, molted to N6 and subsequently to adults, with comparable timing and no observable morphological abnormalities. In a separate experimental group, insects were dissected at N5D4 to assess gene expression levels of *chinmo*, *E93*, *Kr-h1*, and *BR-C* in the wing pads. *chinmo* expression was significantly reduced, and *E93* expression showed a tendency to increase, although the difference compared to controls was not statistically significant. Expression of *Kr-h1* and *BR-C* was not significantly affected (Fig 4B). These findings suggest that the levels of *E93* expression achieved following *chinmo* knockdown were insufficient to trigger adult morphogenesis at the subsequent molt.

### The increase in *E93* expression following dsChinmo treatment occurs gradually

**Fig 5:**
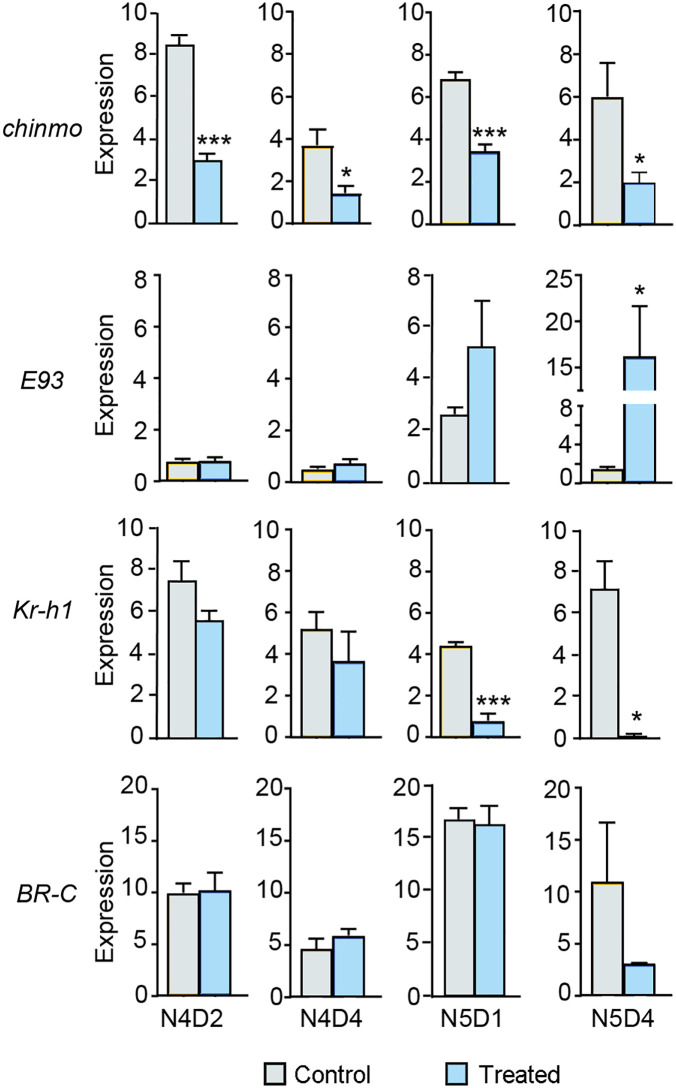
Effects of dsChinmo treatment on the expression of *chinmo* and other genes associated with metamorphosis over time in *Blattella germanica.* dsChinmo was applied in freshly emerged fourth instar female nymphs (N4D0), and gene expression was measured in wing pads at N4D2, N4D4, N5D1, and N5D4. The genes analysed were *chinmo*, *E93*, *Kr-h1*, and *BR-C*. The results are indicated as copies of the examined mRNA per 1000 copies of *Actin-5c* mRNA, and expressed as the mean ± SEM (n = 3-5). Asterisks indicate statistically significant differences with respect to controls (* p < 0.05; *** p < 0.001), calculated with the Student’s *t*-tes*t*.

The time required for *E93* to reach expression levels sufficient to trigger metamorphosis appeared to be a critical factor. To investigate this, we studied the temporal dynamics of *E93* expression after dsChinmo treatment. A total of 6 µg of dsChinmo was injected at N4D0, and the expression levels of *E93*, *chinmo*, *Kr-h1*, and *BR-C* were measured in wing pads at four time points: N4D2, N4D4, N5D1, and N5D4. These were compared to levels in dsMock-treated controls. The results showed that *chinmo* expression was consistently depleted at all time points examined, and that *E93* expression progressively increased over time, although a marked and statistically significant upregulation was only observed at N5D4 (Fig 5), corresponding to nine days post-injection and following one molt. In contrast, *BR-C* expression remained unchanged. Intriguingly, *Kr-h1* expression declined more rapidly than *E93* increased, with a significant reduction already evident by N5D1 (Fig 5). These time-course data suggest that *E93* does not reach the threshold expression levels required to initiate metamorphosis until approximately six to nine days after dsChinmo administration. Consistent with the fact that E93 is a 20E-dependent gene, the dramatic upregulation of *E93* observed at N5D4 (Fig 5) coincides with the peak of ecdysone that occurs that day (Fig 1).

### The reduction in *Kr-h1* expression following Chinmo depletion results from a primary upregulation of *E93*

**Fig 6:**
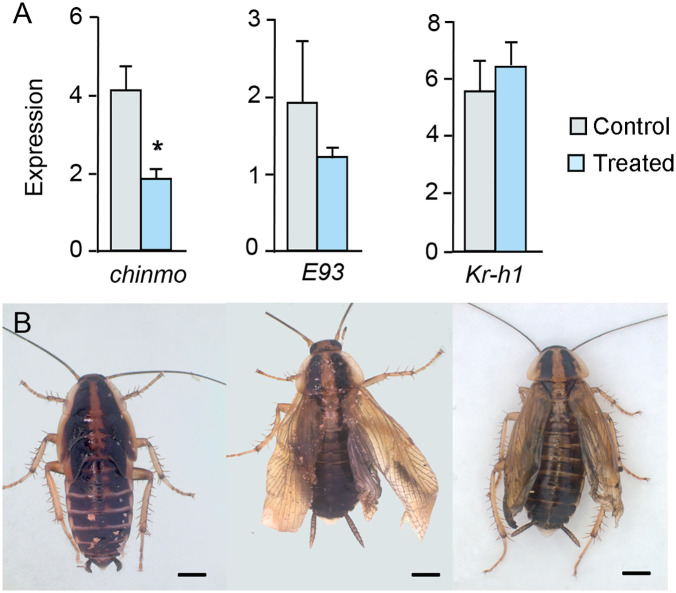
Effects of a double RNAi treatment, dsChinmo + dsE93, on *Blattella germanica* metamorphosis. (A) Effects of dsChinmo + dsE93 treatment on the expression of *chinmo*, *E93*, and *Kr-h1*, measured in wing pads; dsChinmo and dsE93 were applied in freshly emerged fourth instar female nymphs (N4D0), and gene expression was measured at N5D4; the results are indicated as copies of the examined mRNA per 1000 copies of *Actin-5c* mRNA, and expressed as the mean ± SEM (n = 3-5); the asterisk indicates statistically significant differences with respect to controls (p < 0.05) calculated with the Student’s *t*-*t*est. (B) Phenotypes resulting from the dsChinmo + dsE93 treatment; from left to right, supernumerary nymph (N7), adult with not well extended membranous wings and tegmina that emerged from N7, and adult with wrinkled membranous wings and tegmina that emerged from N6; scale bars: 2 mm. Photos: Maria-Dolors Piulachs.

The impact of Chinmo depletion on the expression dynamics of *E93* and *Kr-h1*, along with the known reciprocal repression between these two factors, raises the question of whether Chinmo primarily acts by repressing *E93* or by promoting *Kr-h1* expression. To address this, we conducted a dual RNAi experiment targeting both *chinmo* and *E93* transcripts. The rationale was to offset the upregulation of *E93* normally induced by Chinmo depletion, and then assess the resulting expression of *Kr-h1*. If *Kr-h1* expression remained downregulated, it would suggest that Chinmo activates *Kr-h1* independently of E93. Thus, N4D0 were injected with 6 µg of dsChinmo and 6 µg of dsE93, while control insects received 12 µg of dsMock. Insects were dissected at N5D4, and expression levels of *chinmo*, *E93*, and *Kr-h1* were measured in the wing pads. As expected, *chinmo* expression was significantly depleted. *E93* expression showed a downward trend, though the difference compared to controls was not statistically significant. Importantly, *Kr-h1* expression was similar to controls (Fig 6A). These findings suggest that the primary effect of Chinmo depletion is the upregulation of *E93*, which in turn leads to the downregulation of *Kr-h1*.

To assess the phenotypic outcomes of co-depleting Chinmo and E93, a group of treated insects (n = 10) was monitored through subsequent molts and compared to a control group injected with dsMock (n = 12). All control insects underwent normal development, molting sequentially from N5 to N6 and then to adults. In contrast, the insects treated with dsChinmo and dsE93 exhibited variable developmental trajectories. Four of the treated insects followed the typical molting sequence (N5 - N6 - adult), emerging as normal adults. Three individuals molted from N5 to N6 and subsequently to a supernumerary nymphal instar (N7) (Fig 6B, left), and finally to adults displaying incompletely extended membranous wings and tegmina (Fig 6B, center). The remaining three insects molted to adults directly after N6 but exhibited malformed and wrinkled membranous wings and tegmina (Fig 6B, right). We propose that this phenotypic variability reflects differences in the degree of *E93* knockdown among individuals. The appearance of a supernumerary nymphal instar in three insects likely indicates that *E93* expression was sufficiently depleted to prevent the onset of metamorphosis. In contrast, those that progressed to adulthood may have reached *E93* expression levels above the threshold necessary to initiate metamorphosis, although showing wrinkled membranous wings and tegmina due to apparently nonspecific ecdysis problems.

### Abrupt contributes to the regulation of metamorphosis in *Blattella germanica*

**Fig 7:**
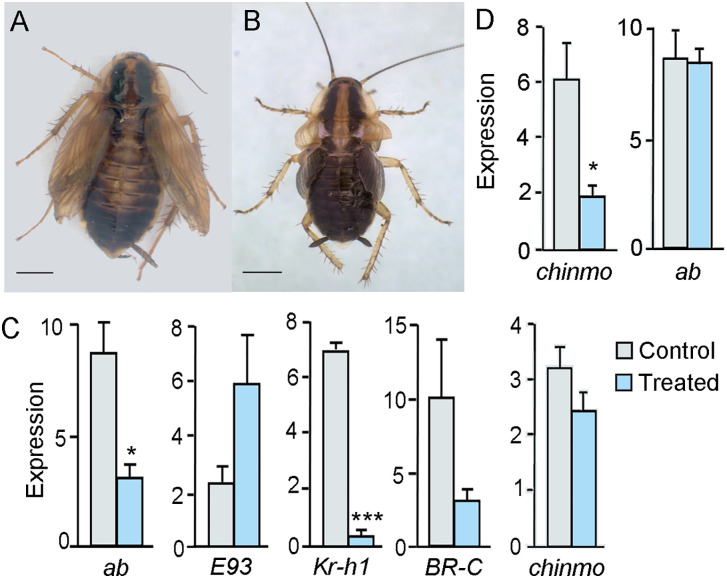
Effects of dsAbrupt treatment on *Blattella germanica* metamorphosis. (A) Adult female with the membranous wings and tegmina not well extended, resulting from the treatment. (B) Precocious adult female resulting from the treatment. (C) Effect of dsAbrupt treatment on the expression of *E93*, *Kr-h1*, *BR-C*, *chinmo*, and *ab* itself. (D) Effects of dsChinmo treatment on *ab* expression. In (C) and (D), dsAbrupt was applied in N4D0, and gene expression was measured at N5D4 in wing pads; the results are indicated as copies of the examined mRNA per 1000 copies of *Actin-5c* mRNA, and expressed as the mean ± SEM (n = 3-4); asterisks indicate statistically significant differences with respect to controls (* p < 0.05; *** p < 0.001), calculated with the Student’s *t*-tes*t*. Photos: Maria-Dolors Piulachs.

Using *ab* sequences from *B. germanica* transcriptomes [18] as queries, we searched for *B. germanica ab* orthologs deposited in GenBank. We identified a partial *ab* sequence (GenBank accession no. PSN37515.1), annotated as a hypothetical protein, which we reannotated as *ab* and subsequently used to design a specific dsAbrupt. We performed RNAi experiments with it following a protocol analogous to that used for *chinmo*. Female nymphs at N4D0 (n = 10) were injected with 6 µg of dsAbrupt, while control insects (n = 12) received 6 µg of dsMock. All insects were maintained through development to monitor subsequent molts. All 12 controls molted normally to N5, then to N6, and finally to adults. Among the dsAbrupt-treated group, 8 insects followed the same molting sequence but emerged as adults with incompletely extended membranous wings and tegmina (Fig 7A). The remaining 2 individuals molted to N5 and then to precocious adults, characterized by externalized membranous wings and tegmina that were short and wrinkled (Fig 7B). In a parallel experiment, the insects were treated identically, but they were dissected at N5D4 to assess gene expression in the wing pads. We measured transcript levels of *ab*, *E93*, *Kr-h1*, *BR-C*, and *chinmo*. The results showed a significant reduction in *ab* and *Kr-h1* expression. That of *E93* and *BR-C* exhibited an increasing and decreasing trend, respectively, although these changes were not statistically significant. Expression of *chinmo* remained practically unchanged (Fig 7C). The emergence of 2 precocious adults among the 10 dsAbrupt-treated insects may be explained by instances in which *E93* expression was sufficiently elevated to trigger adult morphogenesis. Finally, to complete the analysis of regulatory interactions, we examined the effect of Chinmo depletion on *ab* expression. The results showed that *chinmo* knockdown did not significantly affect *ab* transcript levels (Fig 7D).

### Upregulation of *E93* following Abrupt depletion results from reduced *Kr-h1* expression

**Fig 8:**
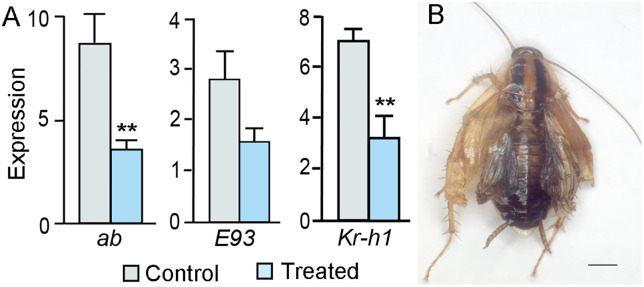
Effects of a double RNAi treatment, dsAbrupt + dsE93, on metamorphosis of *Blattella germanica.* (A) Effects on the expression of metamorphosis genes *ab*, *E93*, and *Kr-h1*, measured in wing pads. dsAbrupt and dsE93 were applied in N4D0, and gene expression was measured at N5D4; the results are indicated as copies of the examined mRNA per 1000 copies of *Actin-5c* mRNA, and expressed as the mean ± SEM (n = 3-5); the two asterisks indicate statistically significant differences with respect to controls (p < 0.01) calculated with the Student’s t-test. (B) Adult with the membranous wings and tegmina not well extended, phenotype resulting from the dsAbrupt + dsE93 treatment; scale bar: 2 mm. Photo: Maria-Dolors Piulachs.

Similar to the case of Chinmo, depletion of Ab led to downregulation of *Kr-h1* and a concomitant increase in *E93* expression. This raises the question of whether the primary effect of Ab is to repress *E93* directly or to promote *Kr-h1* expression, which in turn represses *E93*. To address this, we applied the same experimental approach used for Chinmo. N4D0 females were co-injected with 6 µg of dsAbrupt and 6 µg of dsE93, while control insects received 12 µg of dsMock. Insects were dissected at N5D4 to measure the expression levels of *ab*, *E93*, and *Kr-h1* in wing pads. The results showed significant depletion of *ab* and *Kr-h1* transcripts, while *E93* expression showed a decreasing trend, though not statistically significant compared to controls (Fig 8A). These findings suggest that the primary effect of Ab depletion is the downregulation of *Kr-h1*, which may secondarily permit upregulation of *E93*. To evaluate the phenotypic consequences of the dsAbrupt + dsE93 treatment, a group of treated insects (n = 22) was allowed to continue development and was compared to a control group (n = 14) treated with dsMock. All control insects molted normally from N5 to N6 and then to adults. The insects treated with dsAbrupt and dsE93 also completed these molts, although the resulting adults exhibited wrinkled and incompletely extended membranous wings and tegmina (Fig 8B). This phenotype can be explained because the double treatment resulted in *E93* expression levels that were not low enough to inhibit adult morphogenesis in any experimental insect, although the resulting adults showed wrinkled membranous wings and tegmina attributable to nonspecific ecdysis problems.

## Discussion

### The expression of *chinmo* decreases during the final nymphal instar across various tissues, except in the prothoracic gland

In *B. germanica*, the expression pattern of *chinmo* across different tissues follows a generally parallel trajectory (Fig 1A). Notably, *chinmo* expression consistently declines during the final nymphal instar (N6), as happens in the final larval instar of holometabolan species such as *D. melanogaster* [11,13,14] and *S. frugiperda* [16]. The previously reported *chinmo* expression data in *B. germanica* [14] were presented as fold changes relative to the highest expression level in each tissue, which was arbitrarily set to 1. However, because the highest values were remarkably and differentially elevated, this approach limited the resolution of expression changes at other developmental stages. Our data show that *chinmo* expression in N6 of *B. germanica* generally declines across tissues, except in the PG, where it increases steadily (Fig 1A). This pattern suggests tissue-specific regulation, perhaps linked to PG degeneration, which initiates at the end of N6 [23] and requires the involvement of E93 [24,25]. Interestingly, *E93* expression in the PG during N6 exhibits a distinctive temporal profile: it remains low throughout the instar and only shows a sharp increase on the last day (N6D8) [24]. We hypothesize that the sustained high levels of *chinmo* expression in the PG throughout N6 contribute to the repression of *E93* in that tissue until the end of the instar.

The decline of *chinmo* expression in all other tissues in N6 suggests that JH might have a stimulatory effect, since the concentration of this hormone declines at that instar (Fig 1B). However, application of Methoprene at N6 did not enhance *chinmo* expression (Fig 1C). We can also consider the hypothesis that the increase in E93 that occurs at N6 [24] might be responsible for the *chinmo* expression decline in this instar. However, the reduction in *E93* expression levels indirectly caused by Methoprene application did not affect *chinmo* expression (Fig 1C). We can therefore suggest that the downregulation of *chinmo* in N6 of *B. germanica* is not directly due to a decrease in JH levels nor to an increase in E93.

In other species, the mechanisms regulating *chinmo* expression are also unclear. In *T. castaneum*, the application of the Methoprene to freshly emerged final-instar larvae resulted in increased *chinmo* expression. In contrast, knockdown of key genes associated with JH biosynthesis (such as *JH acid O-methyltransferase*) or JH signaling (including *Methoprene-tolerant* and *Kr-h1*) during the fifth larval instar did not alter *chinmo* expression [15]. In *S. frugiperda* Sf9 cells, Methoprene treatment alone did not affect *chinmo* expression, whereas 20E increased it by approximately 1.4-fold. Combined treatment with Methoprene and 20E resulted in a 2.6-fold increase in *chinmo* expression [16]. The available data suggest that *chinmo* could be regulated differently in different tissues and stages, and even in hemimetabolan and holometabolan species.

### Chinmo depletion causes precocious metamorphosis in hemimetabolans

The fact that Chinmo depletion in nymphs induces precocious metamorphosis in *B. germanica* indicates its role in maintaining the nymphal state. All insects treated with dsChinmo at N4 molted to N5 and then directly to adults, skipping the N6 instar. These precocious adults resembled those previously described [14]. However, in that study, only 43% of treated individuals displayed this phenotype. This discrepancy may reflect lower RNAi efficiency in that experimental context. Nonetheless, the extent of transcript depletion was not reported, leaving this interpretation uncertain. Since precocious adults skipped the N6 instar, it is unsurprising that they were smaller than control adults. However, when wing size is considered relative to overall body size, it becomes apparent that the wings of precocious adults are disproportionately shorter compared to those of control adults. This observation suggests that proper and proportional wing development requires a minimum growth period, which is not achieved during the precocious metamorphosis induced by Chinmo depletion. In holometabolans, Chinmo similarly maintains the larval state. Yet, due to the two-stage metamorphic transition (larva–pupa–adult), *chinmo* silencing typically results in larvae showing only subtle or no visible signs of precocious metamorphosis. Thus, *chinmo* silencing in *D. melanogaster* [11,14] and *T. castaneum* [15] led to larval phenotypes with minor pupal or adult traits, while no morphological changes were observed in *S. frugiperda* [16].

Our examination of the ovaries from precocious adults of *B. germanica* revealed a distinct phenotype in which the follicular epithelium only partially covered the oocyte of the BOF. This observation raised the question of whether the phenotype resulted from insufficient time for full development due to precocious metamorphosis, or from a specific role of Chinmo in regulating follicular cell proliferation and growth. We found that the expression levels of genes belonging to the Notch and Hippo pathways in ovaries from Chinmo-depleted nymphs were comparable to those in controls. Furthermore, dsChinmo treatment in newly emerged adults did not significantly affect ovarian development, particularly that of the follicular epithelium. Altogether, these results indicate that the incomplete development of the follicular epithelium in precocious adults is attributable to insufficient time for proper growth, similar to the case of the membranous wings and tegmina discussed above.

### Chinmo and Abrupt contribute to maintaining the nymphal state by repressing *E93* and stimulating *Kr-h1*, respectively

Our results indicate that Chinmo contributes to the maintenance of the nymphal state in *B. germanica* by repressing *E93*. Chinmo depletion increases *E93* expression, which in turn downregulates *Kr-h1*, consistent with the MEKRE93 pathway. Notably, Chinmo does not significantly affect *BR-C* expression in this species, an important distinction from holometabolan insects. In holometabolans, *chinmo* silencing also elevates *E93* expression, but interpretation is complicated by its interaction with *BR-C*. In *D melanogaster*, *chinmo* silencing increases *E93* expression in larval tissues and *BR-C* expression in imaginal discs [11]. A subsequent study [14] indicates that *BR-C* is upregulated in both tissues. In *T. castaneum* [15] and *S. frugiperda* [16], Chinmo depletion increases both *BR-C* and *E93*, as shown in whole-body and cell line experiments, respectively.

The precocious adults resulting from Chinmo depletion in *B. germanica* resemble those obtained by depleting JH pathway transducers like Kr-h1 [26]. However, while dsKr-h1 treatment at N5 triggers precocious metamorphosis at the next molt, dsChinmo applied at the same instar does not. This difference is likely due to the slower induction of *E93* following Chinmo depletion, suggesting distinct mechanisms of action of Kr-h1 and Chinmo. In the silkworm *Bombyx mori*, Kr-h1 directly binds the *E93* promoter and inhibits its expression [27], whereas in *S. littoralis*, Chinmo appears to act via chromatin accessibility [16], potentially explaining the slower effect. Our RNAi data also show that Ab contributes to maintaining the nymphal state by upregulating *Kr-h1*, thus reinforcing the effect of JH. Together, these functions sustain the maintenance of the nymphal state. In *T. castaneum*, Chinmo and Ab have synergistic effects on the regulation of metamorphosis, but Ab depletion does not affect *Kr-h1* expression [15].

### The regulation of insect metamorphosis is more complex than previously thought

**Fig 9:**
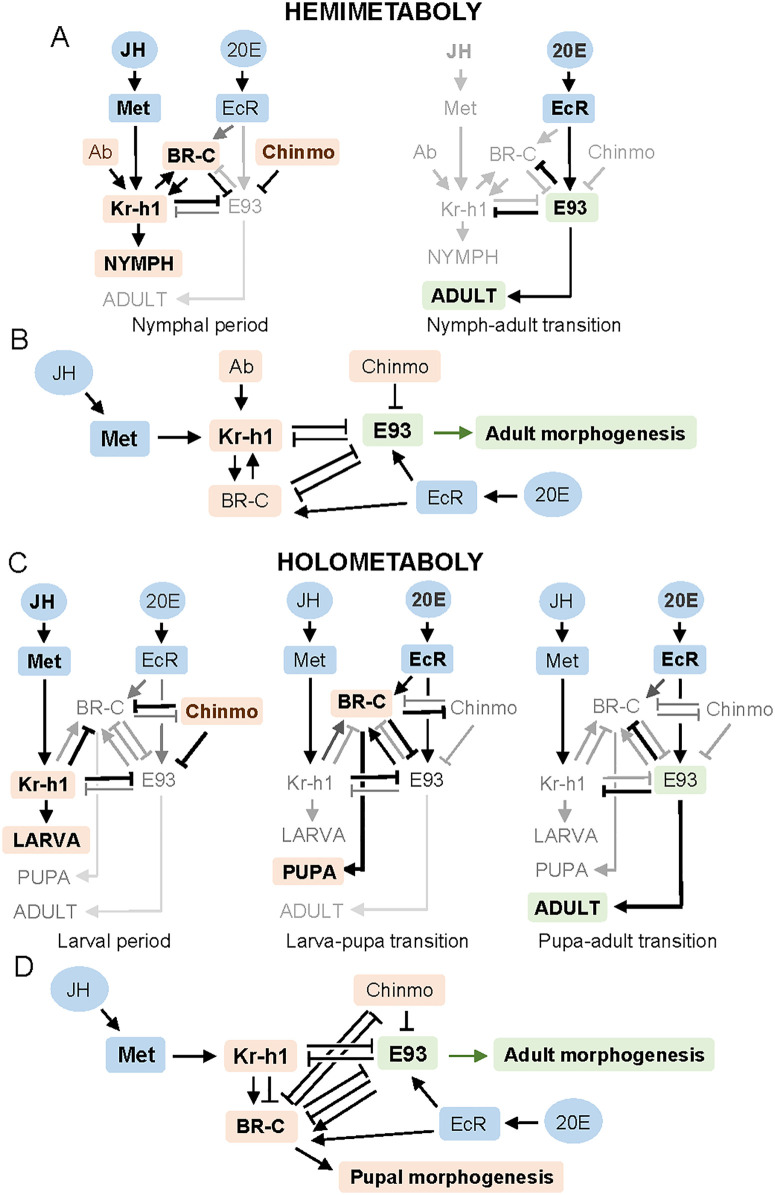
Chinmo and Ab in the MEKRE93 pathway. (A) Interactions between Kr-h1, E93, BR-C, Chinmo and Ab operating during the nymphal period (left) and in the transition from the final nymphal instar to the adult (right). (B) The MEKRE93 pathway in hemimetabolan insects, including the action of Chinmo and Ab. (C) Interactions between Kr-h1, E93, BR-C and Chinmo operating during the larval period (left), in the transition to the pupal stage (middle), at from the pupa to the adult (right); in (A) and (C), *Kr-h1* expression is promoted by JH through its receptor Met whereas that of *BR-C* and *E93* is promoted by 20E, through its receptor EcR. (D) The MEKRE93 pathway in holometabolan insects, including the action of Chinmo. The schemes aggregate the interactions reported so far, based on data obtained in *Tribolium castaneum*, *Bombyx mori*, and *Drosophila melanogaster* by various authors, as reviewed in [1]. The repressive effect of BR-C on *chinmo* has been reported in *D. melanogaster* [13], that of Chinmo on *BR-C* and *E93* has been reported in *D. melanogaster*, *T. castaneum*, and *Spodoptera frugiperda* [11,14–16], and that of BR-C on *E93* has been reported in *Gryllus bimaculatus* [37] and *S. frugiperda* [30]. Depletion of Ab in *T. castaneum* did not alter the expression of *BR-C*, *E93*, and *Kr-h1* [15]. Thus, the mechanism of action of Ab associated with metamorphosis in holometabolans remains unknown.

The regulation of hemimetabolan metamorphosis has been explained by the essential MEKRE93 pathway: JH induces Kr-h1, which represses *E93*; once JH vanishes in the final nymphal instar, Kr-h1 declines, *E93* is upregulated, and metamorphosis proceeds [9]. Our results on *B. germanica* now show that Chinmo plays a critical role in maintaining the nymphal state by repressing *E93*, acting alongside Kr-h1. Moreover, Ab enhances *Kr-h1* expression, bolstering the antimetamorphic effect of JH (Fig 9A). Additionally, we have also verified that Chinmo and Ab do not interact with each other (Fig 9B).

In holometabolans, the roles of Kr-h1, BR-C, and E93 as main players in the larval, pupal, and adult stages, respectively, are well established (reviewed in [1]). More recent data indicate that Chinmo contributes to maintaining the larval state in *D. melanogaster*, *T. castaneum*, and *S. frugiperda* [11,14–16]. In *T. castaneum*, Ab cooperates with Chinmo in repressing metamorphosis [15], increasing the complexity of the MEKRE93 pathway in holometabolans. Moreover, BR-C regulation introduces additional intricacies. In early larval instars, JH represses *BR-C*, but after pupal commitment, it promotes *BR-C* expression [6]. In *T. castaneum*, *Kr-h1* expression remains active until the final larval instar, when it drops briefly, allowing a modest increase in *E93* and a strong rise in *BR-C*, initiating pupation. Later, in the pupa, *Kr-h1* and *BR-C* respective expressions decline, while that of *E93* surges, triggering adult morphogenesis [2,28,29]. These dynamics are summarized in Fig 9C, based mainly on studies in *T. castaneum*, *B. mori*, and *D. melanogaster* (reviewed in [1]). The repressive effect of BR-C on *E93* has been recently reported in *S. frugiperda* [30]. Fig 9D integrates these interactions into the MEKRE93 pathway associated with holometabolan metamorphosis.

### New insights into the evolution of insect metamorphosis

Recent reviews on insect metamorphosis [1,31] agree that holometaboly evolved from hemimetabolan ancestors, likely under the selective pressure of concealing wing primordia during embryogenesis and then along juvenile stages. The larva of the last common ancestor of holometabolans (Endopterygota) likely resembled a hemimetabolan nymph in overall body plan, with the key difference being that wing primordia were internalized as imaginal precursor cells during embryogenesis. Then, precursor cells for other adult structures were also internalized, resulting in increasingly divergent larval forms across endopterygote lineages. Subsequently, during the pupal stage, the adult body plan generally would develop from these imaginal precursors (reviewed in [1]).

At the regulatory level, the transition to holometaboly must have involved changes during embryogenesis that allowed the internal segregation of imaginal cells. Postembryonically, the juvenile-maintaining roles of Kr-h1, Chinmo, and Ab, and the adult-specifying role of E93, were already present in hemimetabolans and conserved in holometabolans. While Chinmo represses both *BR-C* and *E93* in holometabolans [11,14–16], our data show that it represses *E93* but not *BR-C* in the hemimetabolan *B. germanica*. Thus, Chinmo repression of *E93* was conserved, whereas its inhibitory effect on *BR-C* should have evolved in holometabolans. Our findings also show that Ab enhances *Kr-h1* expression in *B. germanica*, but this is not the case in *T. castaneum* [15].

The evolution of BR-C function is particularly striking: it expanded from regulating wing development in hemimetabolan nymphs to orchestrating pupal morphogenesis in holometabolan larvae. Moreover, *BR-C* is stimulated by JH via *Kr-h1* in hemimetabolan nymphs [32,33], while in holometabolan larvae, JH inhibits *BR-C* [34]. Thus, the evolution of holometaboly involved a change in the action of JH over *BR-C*, from stimulatory to inhibitory, during the larval period [32]. However, the stimulatory effect of JH on *BR-C* is retained, transiently, in the pupa [35]. The E93-mediated activation of *BR-C* in *T. castaneum* prepupae [28] may represent a novel regulatory element that emerged with holometaboly.

**Fig 10:**
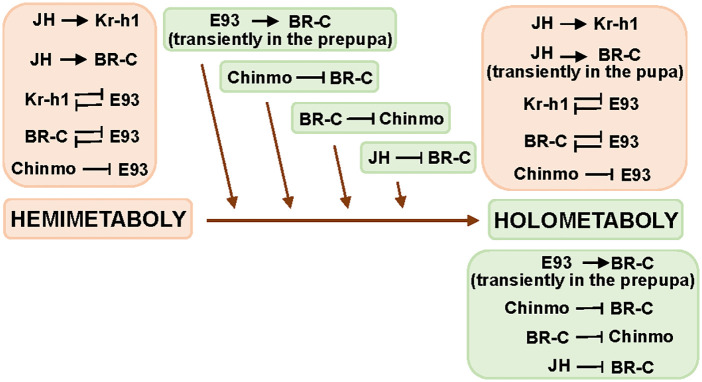
Conserved (orange) and new (green) interactions involved in the evolutionary transition from hemimetaboly to holometaboly. The context of the interactions shown herein is detailed in Fig 9.

Fig 10 summarizes the interactions between regulatory factors conserved in hemimetabolans and holometabolans, as well as those acquired during the evolution of holometaboly. The high degree of conservation is notable, even if mechanistic details may differ between groups. Still, the expanded regulatory network now emerging suggests that metamorphosis, and its evolutionary history, is more intricate than previously envisioned and likely more complex than current models, like that presented in this paper, capture.

## Materials and methods

### Insects

*B. germanica* specimens used in the experiments and observations were obtained from a colony fed *ad libitum* on Panlab 125 dog food, water, and reared in the dark at 30 ± 1ºC and 60–70% r.h. They were carbon dioxide-anaesthetized before dissections and tissue sampling.

### Identification of *chinmo* and *abrupt* genes in *Blattella germanica*

Using annotated sequences from *B. germanica* transcriptomes [18] as queries, we searched *B. germanica chinmo* and *ab* orthologs deposited in GenBank. We found a partial, short sequence of *chinmo* (GenBank: PSN45666.1, annotated as “hypothetical protein C0J52_22365”, Harrison et al., 2018) that was completed and deposited to the GenBank with the accession number: PV101163. Using the program BLAST, we found a sequence for *ab* (GenBank: PSN37515.1, annotated as “hypothetical protein C0J52_26295”, Harrison et al., 2018), that was reannotated as *B. germanica abrupt*.

### RNA extraction and retrotranscription to cDNA

All RNA extractions were carried out with the Tissue Total RNA purification Kit (Canvax Biotech). An amount of 400 ng from each RNA extraction was treated with DNase (Promega) and reverse transcribed with the Transcriptor First Strand cDNA Synthesis Kit (Roche). RNA quantity and quality were estimated by spectrophotometric absorption at 260 nm in a Nanodrop Spectrophotometer (MicroDigital Co, Ltd).

### Determination of mRNA levels with quantitative real-time PCR

Quantitative real-time PCR (qRT-PCR) reactions were carried out in an iQ5 Real-Time PCR Detection System (Bio-Rad Laboratories), using iTaq Universal SYBR Green Supermix (Bio-Rad Laboratories). A control without template was included in all batches. The primers used for each transcript measured are detailed in S1 Table. The efficiency of each primer set was first validated by constructing a standard curve through four serial dilutions. mRNA levels were calculated using the Bio-Rad CFX Maestro (Version 5.3.022.1030). The amplification reactions were performed at 95°C for 3 min, 44 cycles of 95°C for 10 s plus 57°C for 1 min, followed by 95°C for 10 s, and finally, the melting curve: from 57°C to 95°C with a measurement at each 0.5°C increase. Expression levels were calculated using the 2-ΔΔCt method [36], and relative to *Actin-5c* expression, whose expression profiles do not present variations throughout the periods studied. Results are given as copies of mRNA per 1,000 copies of *Actin-5c* mRNA.

### RNA interference

dsRNAs were synthesized using RiboMAX Large Scale RNA Production System T7 (Promega) following manufacturer instructions. RNAi experiments were used to target *chinmo*, *ab*, and *E93* transcripts. The primers used to generate templates by PCR for transcription of these dsRNAs are detailed in S1 Table. The fragments were amplified by PCR and cloned into the pSTBlue-1 vector (Novagen). In all cases, we used a sequence from *Autographa californica* nucleopolyhydrosis virus (accession number K01149) as control dsRNA (dsMock). A volume of 1 µL of each dsRNA solution (6 µg/µL) was injected into the abdomen of specimens at chosen ages and stages with a Hamilton 75N syringe. Control specimens were treated with the same dose and volume of dsMock. In the case of double treatments, 6 µg of each of the two dsRNAs, and 12 µg of dsMock were injected in a volume of 1 µL.

### Treatments with methoprene

A solution of 15 µg per µL of Methoprene (Sigma**-**Aldrich) in acetone was used. A volume of 2 µL of this solution was applied topically on the dorsal part of the abdomen of N6.

### Morphological studies and imaging.

The treated and control insects were examined and photographed using a stereomicroscope Zeiss DiscoveryV8. Biometrical measurements of oocyte lengths were carried out with an ocular micrometer adapted to this stereomicroscope. To examine the ovaries of precocious adults compared with controls, ovaries were dissected and immediately fixed in paraformaldehyde (4% in PBS 0.2 M; pH 6.8) for 2 h. Washed for 10 min in PBT (PBS 0.2M; pH 6.8 + 0.2% Tween 20) [22] three times, and stained for 20 min in 300 ng/mL of Phalloidin-TRITC (tetramethylrhodamine isothiocyanate, Merck), washed two times in PBT (10 min each). Then, the ovaries were incubated for 5 min in 1μg/mL of DAPI (4,6-diamidino-2-phenylindole, Merck) in PBT, and washed again twice in PBT. Samples were incubated in 1.5 μg/mL of wheat germ agglutinin (WGA) for 2 min to stain the cell membranes, and washed again. Finally, ovaries were then mounted in Mowiol (Calbiochem) and observed using a Zeiss AxioImager Z1 fluorescence microscope (Apotome) (Carl Zeiss MicroImaging).

### Statistics

Quantitative data are expressed as mean ± standard error of the mean (SEM). In qRT-PCR determinations, statistical analyses between groups were tested by GraphPad Prism version 8.1.0 for Windows, GraphPad Software. Data were analyzed for homogeneity and normality of variance using the Shapiro–Wilk test, which indicated that no transformations were needed. All datasets passed the normality test. Significant differences between control and treated groups were calculated using the *Student’s t*-test.

## Supporting information

S1 Fig Expression of genes associated with Notch (N) and Hippo (Hpo) pathways in the ovaries of precocious adults triggered by Chinmo depletion, compared with control females of *Blattella germanica.* (TIFF)

S2 Fig Ovarian effects of dsChinmo treatment in newly emerged adult females of *Blattella germanica.* (TIFF)

S1 Table Primer sequences for quantifying different gene expression (qRT-PCR) and to generate dsRNAs. (TIFF)

## References

[1] Belles X. Insect metamorphosis. From natural history to regulation of development and evolution. London: Academic Press; 2020.

[2] Minakuchi C, Namiki T, Shinoda T. Krüppel homolog 1, an early juvenile hormone-response gene downstream of Methoprene-tolerant, mediates its anti-metamorphic action in the red flour beetle *Tribolium castaneum*. Dev Biol. 2009;325(2):341–50. doi: 10.1016/j.ydbio.2008.10.016 19013451

[3] Minakuchi C, Zhou X, Riddiford LM. Krüppel homolog 1 (Kr-h1) mediates juvenile hormone action during metamorphosis of *Drosophila melanogaster*. Mech Dev. 2008;125(1–2):91–105. doi: 10.1016/j.mod.2007.10.002 18036785 PMC2276646

[4] Kiss I, Beaton AH, Tardiff J, Fristrom D, Fristrom JW. Interactions and developmental effects of mutations in the Broad-Complex of *Drosophila melanogaster*. Genetics. 1988;118(2):247–59. doi: 10.1093/genetics/118.2.247 3129334 PMC1203278

[5] Karim FD, Guild GM, Thummel CS. The *Drosophila* Broad-Complex plays a key role in controlling ecdysone-regulated gene expression at the onset of metamorphosis. Development. 1993;118(3):977–88. doi: 10.1242/dev.118.3.977 8076529

[6] Zhou B, Hiruma K, Shinoda T, Riddiford LM. Juvenile hormone prevents ecdysteroid-induced expression of broad complex RNAs in the epidermis of the tobacco hornworm, *Manduca sexta*. Dev Biol. 1998;203(2):233–44. doi: 10.1006/dbio.1998.9059 9808776

[7] Ureña E, Manjón C, Franch-Marro X, Martín D. Transcription factor E93 specifies adult metamorphosis in hemimetabolous and holometabolous insects. Proc Natl Acad Sci U S A. 2014;111(19):7024–9. doi: 10.1073/pnas.1401478111 24778249 PMC4024875

[8] Truman JW, Riddiford LM. Endocrine insights into the evolution of metamorphosis in insects. Annu Rev Entomol. 2002;47:467–500. doi: 10.1146/annurev.ento.47.091201.145230 11729082

[9] Belles X, Santos CG. The MEKRE93 (Methoprene tolerant-Krüppel homolog 1-E93) pathway in the regulation of insect metamorphosis, and the homology of the pupal stage. Insect Biochem Mol Biol. 2014;52:60–8. doi: 10.1016/j.ibmb.2014.06.009 25008785

[10] Belles X. Krüppel homolog 1 and E93: The doorkeeper and the key to insect metamorphosis. Arch Insect Biochem Physiol. 2020;103(3):e21609. doi: 10.1002/arch.21609 31385626

[11] Truman JW, Riddiford LM. *Chinmo* is the larval member of the molecular trinity that directs Drosophila metamorphosis. Proc Natl Acad Sci U S A. 2022;119(15):e2201071119. doi: 10.1073/pnas.2201071119 35377802 PMC9169713

[12] Zhu S, Lin S, Kao C-F, Awasaki T, Chiang A-S, Lee T. Gradients of the Drosophila *Chinmo* BTB-zinc finger protein govern neuronal temporal identity. Cell. 2006;127(2):409–22. doi: 10.1016/j.cell.2006.08.045 17055440

[13] Narbonne-Reveau K, Maurange C. Developmental regulation of regenerative potential in Drosophila by ecdysone through a bistable loop of ZBTB transcription factors. PLoS Biol. 2019;17(2):e3000149. doi: 10.1371/journal.pbio.3000149 30742616 PMC6386533

[14] Chafino S, Giannios P, Casanova J, Martín D, Franch-Marro X. Antagonistic role of the BTB-zinc finger transcription factors Chinmo and Broad-Complex in the juvenile/pupal transition and in growth control. Elife. 2023;12:e84648. doi: 10.7554/eLife.84648 37114765 PMC10234633

[15] Khong H, Hattley KB, Suzuki Y. The BTB transcription factor, Abrupt, acts cooperatively with Chronologically inappropriate morphogenesis (Chinmo) to repress metamorphosis and promotes leg regeneration. Dev Biol. 2024;509:70–84. doi: 10.1016/j.ydbio.2024.02.006 38373692

[16] Chen X, Koo J, Kumar Arya S, Palli SR. Chronologically inappropriate morphogenesis (Chinmo) is required for maintenance of larval stages of fall armyworm. Proc Natl Acad Sci U S A. 2024;121(49):e2411286121. doi: 10.1073/pnas.2411286121 39589873 PMC11626174

[17] Harrison MC, Jongepier E, Robertson HM, Arning N, Bitard-Feildel T, Chao H, et al. Hemimetabolous genomes reveal molecular basis of termite eusociality. Nat Ecol Evol. 2018;2(3):557–66. doi: 10.1038/s41559-017-0459-1 29403074 PMC6482461

[18] Ylla G, Piulachs M-D, Belles X. Comparative transcriptomics in two extreme neopterans reveals general trends in the evolution of modern insects. iScience. 2018;4:164–79. doi: 10.1016/j.isci.2018.05.017 30240738 PMC6147021

[19] Treiblmayr K, Pascual N, Piulachs M-D, Keller T, Belles X. Juvenile hormone titer versus juvenile hormone synthesis in female nymphs and adults of the German cockroach, *Blattella germanica*. J Insect Sci. 2006;6:1–7. doi: 10.1673/031.006.4301 20233097 PMC2990300

[20] Cruz J, Martín D, Pascual N, Maestro JL, Piulachs MD, Bellés X. Quantity does matter. Juvenile hormone and the onset of vitellogenesis in the German cockroach. Insect Biochem Mol Biol. 2003;33(12):1219–25. doi: 10.1016/j.ibmb.2003.06.004 14599494

[21] Ylla G, Belles X. Towards understanding the molecular basis of cockroach tergal gland morphogenesis. A transcriptomic approach. Insect Biochem Mol Biol. 2015;63:104–12. doi: 10.1016/j.ibmb.2015.06.008 26086932

[22] Irles P, Piulachs M-D. Unlike in drosophila meroistic ovaries, hippo represses notch in *Blattella germanica* panoistic ovaries, triggering the mitosis-endocycle switch in the follicular cells. PLoS One. 2014;9(11):e113850. doi: 10.1371/journal.pone.0113850 25426635 PMC4245235

[23] Romaña I, Pascual N, Belles X. The ovary is a source of circulating ecdysteroids in *Blattella germanica*. Eur J Entomol. 1995;93:93–103.

[24] Kamsoi O, Belles X. E93-depleted adult insects preserve the prothoracic gland and molt again. Development. 2020;147(22):dev190066. doi: 10.1242/dev.190066 33077428

[25] Zeng M, Yan Z-Y, Lv Y-N, Zeng J-M, Ban N, Yuan D-W, et al. Molecular basis of E93-dependent tissue morphogenesis and histolysis during insect metamorphosis. Insect Biochem Mol Biol. 2025;177:104249. doi: 10.1016/j.ibmb.2024.104249 39674518

[26] Lozano J, Belles X. Conserved repressive function of Krüppel homolog 1 on insect metamorphosis in hemimetabolous and holometabolous species. Sci Rep. 2011;1:163. doi: 10.1038/srep00163 22355678 PMC3240953

[27] Kayukawa T, Jouraku A, Ito Y, Shinoda T. Molecular mechanism underlying juvenile hormone-mediated repression of precocious larval-adult metamorphosis. Proc Natl Acad Sci U S A. 2017;114(5):1057–62. doi: 10.1073/pnas.1615423114 28096379 PMC5293048

[28] Chafino S, Ureña E, Casanova J, Casacuberta E, Franch-Marro X, Martín D. Upregulation of E93 gene expression acts as the trigger for metamorphosis independently of the Threshold Size in the Beetle *Tribolium castaneum*. Cell Rep. 2019;27(4):1039-1049.e2. doi: 10.1016/j.celrep.2019.03.094 31018122

[29] Ureña E, Chafino S, Manjón C, Franch-Marro X, Martín D. The occurrence of the holometabolous pupal stage requires the interaction between E93, Krüppel-Homolog 1 and Broad-Complex. PLoS Genet. 2016;12(5):e1006020. doi: 10.1371/journal.pgen.1006020 27135810 PMC4852927

[30] Liu C, Wu M-Z, Zheng Z-J, Fan S-T, Tan J-F, Jiao Y, et al. Knockout BR-C induces premature expression of E93 thus triggering adult differentiation under larval morphology. Pest Manag Sci. 2025;81(4):1923–33. doi: 10.1002/ps.8592 39641237

[31] Truman JW. The Evolution of Insect Metamorphosis. Curr Biol. 2019;29(23):R1252–68. doi: 10.1016/j.cub.2019.10.009 31794762

[32] Huang J-H, Lozano J, Belles X. Broad-complex functions in postembryonic development of the cockroach *Blattella germanica* shed new light on the evolution of insect metamorphosis. Biochim Biophys Acta. 2013;1830(1):2178–87. doi: 10.1016/j.bbagen.2012.09.025 23041750

[33] Konopova B, Jindra M. Broad-Complex acts downstream of Met in juvenile hormone signaling to coordinate primitive holometabolan metamorphosis. Development. 2008;135(3):559–68. doi: 10.1242/dev.016097 18171683

[34] Konopova B, Smykal V, Jindra M. Common and distinct roles of juvenile hormone signaling genes in metamorphosis of holometabolous and hemimetabolous insects. PLoS One. 2011;6(12):e28728. doi: 10.1371/journal.pone.0028728 22174880 PMC3234286

[35] Zhou X, Riddiford LM. Broad specifies pupal development and mediates the “status quo” action of juvenile hormone on the pupal-adult transformation in *Drosophila* and *Manduca*. Development. 2002;129(9):2259–69. doi: 10.1242/dev.129.9.2259 11959833

[36] Livak KJ, Schmittgen TD. Analysis of relative gene expression data using real-time quantitative PCR and the 2(-Delta Delta C(T)) Method. Methods. 2001;25(4):402–8. doi: 10.1006/meth.2001.1262 11846609

[37] Ishimaru Y, Tomonari S, Watanabe T, Noji S, Mito T. Regulatory mechanisms underlying the specification of the pupal-homologous stage in a hemimetabolous insect. Philos Trans R Soc Lond B Biol Sci. 2019;374(1783):20190225. doi: 10.1098/rstb.2019.0225 31438810 PMC6711289

