# Supplementary figures and images for: Chinmo function in cockroaches provides new insights into the regulation and evolution of insect metamorphosis
Source: PLoS Genet. 2025 Dec 26;21(12):e1011993. doi: 10.1371/journal.pgen.1011993 (PMC12758806; doi:10.1371/journal.pgen.1011993)

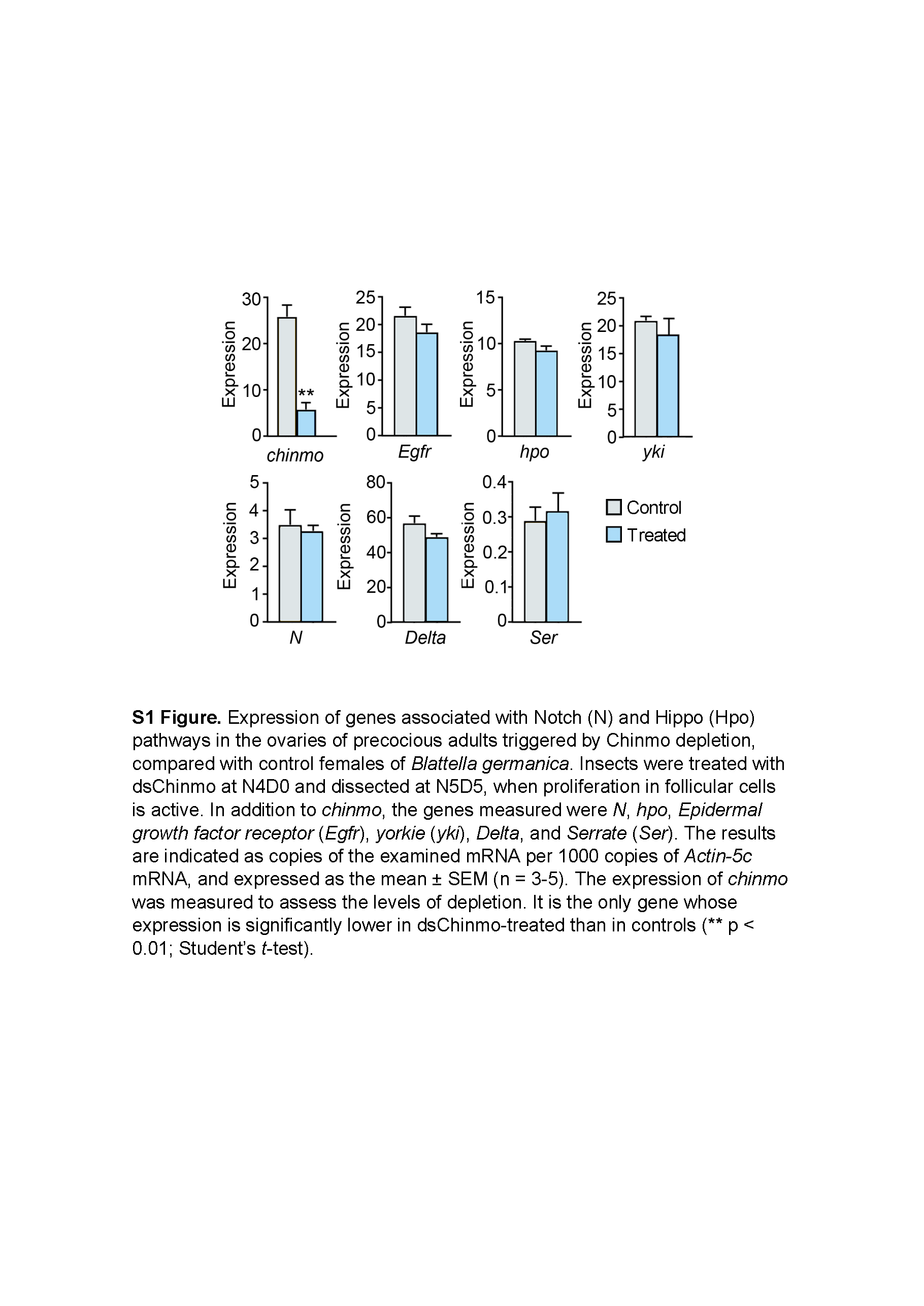

Supplement: S1 Fig — (TIFF) [file pgen.1011993.s001.tiff]

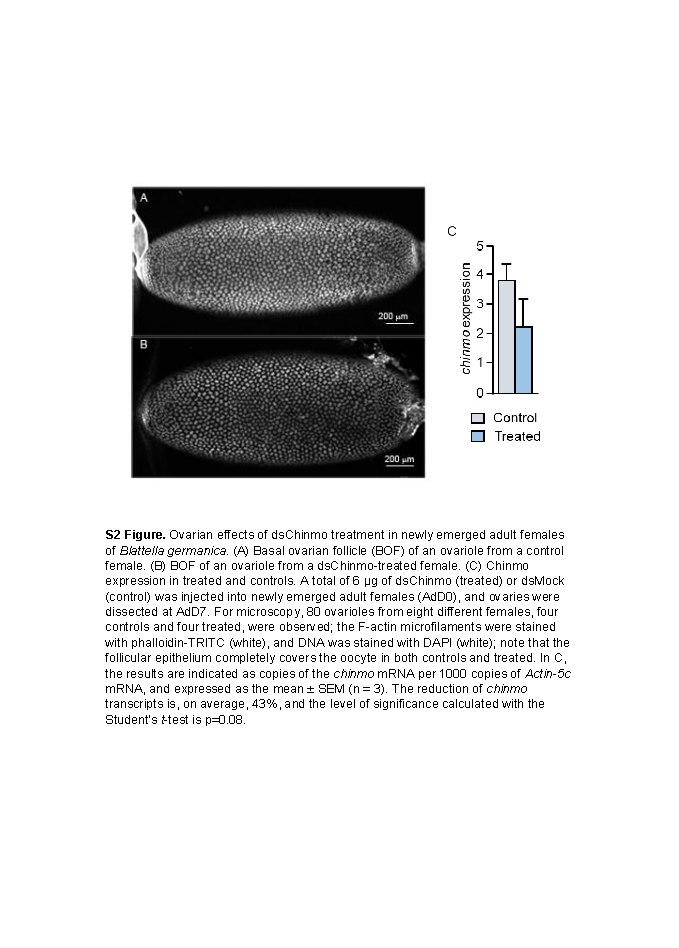

Supplement: S2 Fig — (TIFF) [file pgen.1011993.s002.tiff]
